# Transcriptome Sequencing Analysis of lncRNA and mRNA Expression Profiles in Bone Nonunion

**DOI:** 10.1155/2022/9110449

**Published:** 2022-10-12

**Authors:** Jian Zhou, Rongjun Wan, Qunyan Tian, Ziyi Wu, Zhengjun Lin, Wanchun Wang, Cheng Tao, Tang Liu

**Affiliations:** ^1^Department of Orthopedics, The Second Xiangya Hospital of Central South University, Changsha, Hunan 410011, China; ^2^Department of Respiratory Medicine, National Key Clinical Specialty, Branch of National Clinical Research Center for Respiratory Disease, Xiangya Hospital, Central South University, Changsha, Hunan 410008, China; ^3^National Clinical Research Center for Geriatric Disorders, Xiangya Hospital, Changsha, Hunan 410008, China

## Abstract

**Background:**

Bone nonunion is a serious complication of fracture. This study explored the differentially expressed lncRNAs (DELs) and mRNAs (DEGs) and identified potential lncRNA-mRNA interactions in bone nonunion.

**Methods:**

We extracted total RNA from three bone nonunion and three bone union patient tissue samples. RNA sequencing was performed to detect DELs and DEGs between bone nonunion and union tissue samples. The lncRNAs and genes with absolute log2-fold change (log2FC) > 1 and adjusted *p* value < 0.05 were further chosen for gene ontology (GO) and Kyoto Encyclopedia of Genes and Genomes (KEGG) enrichment analysis. lncRNA and targeted mRNA interaction networks were constructed.

**Results:**

We observed 179 DELs and 415 DEGs between the bone nonunion and union tissue samples. GO analysis indicated that DELs and DEGs were mainly enriched in the chondroitin sulfate proteoglycan biosynthetic process. DELs and DEGs were enriched in “ECM-receptor interaction” and “*Staphylococcus aureus* infection” KEGG pathways. Several potential lncRNA-mRNA interactions were also predicted.

**Conclusions:**

This study identified bone nonunion-associated lncRNAs and mRNAs using deep sequencing that may be useful as potential biomarkers for bone nonunion.

## 1. Introduction

Bone nonunion, a serious complication of fracture, occurs in approximately 5–10% of patients with bone fractures [1–7]. Infected bone nonunion is caused by many factors, including fractures and accidents. Bone nonunion may lead to delayed union and amputation, which further contributes to functional limitation, disability, and poor quality of life [1, 2]. The most common causes of bone nonunion include infection, insufficient local blood supply, separation of fracture ends, and insufficient fracture stabilization [8, 9]. The use of antibiotics has improved the treatment of bone infections, but bone nonunion remains an obstacle in the repair of damaged bone [3, 4].

Currently, nonunion is a serious challenge in the treatment of bone loss associated with bone infections. The process of bone remodeling includes the breakdown and resorption of bone and the formation of new bone. Osteoclasts are responsible for bone breakdown and resorption, whereas osteoblasts are responsible for new bone formation. Osteoclasts attach to the older bone area, secrete acidic substances to dissolve minerals, secrete protease to digest the bone matrix, and form bone resorption lacuna. Subsequently, osteoblasts migrate to the resorbed site and secrete the bone matrix that is then mineralized to form new bone. The balance between osteoclastic and osteogenic processes is substantial in maintaining the normal bone mass. However, this balance is compromised because resorption replaces formation in case of bone infection, resulting in bone loss or bone nonunion [10]. A previous study indicated that differentially expressed miRNAs might be a potential diagnostic and therapeutic biomarker for infected tibial nonunion [11]. Additionally, the data from the GEO dataset indicated that ADAMTS18 and TGFBR3 genes were differentially expressed in nonunion skeletal fracture [12]. Moreover, the coinjection of BMP and DCN into the bone nonunion area could improve the induction of bone formation [13, 14]. However, the exact molecular mechanisms underlying bone nonunion remain unclear at present. Therefore, it is critical to explore the etiological mechanism of bone nonunion and to develop new targets for the diagnosis and treatment of infected bone nonunion.

Long noncoding RNAs (lncRNAs) are a class of RNAs longer than 200 nucleotides. Previous studies have indicated that lncRNAs can act as master regulators, affecting target gene expression levels [15]. Growing evidence suggests that lncRNAs are involved in the epigenetic regulation of gene expression, transcription, cell death, and other important biological processes [16, 17]. Previous reports showed that many lncRNAs are related to osteoclast and osteoblast cell functions. For instance, lncGHET1, lncRhno1, lncTUG1, and lncUCA1 are identified to be involved with osteoblast proliferation and differentiation [18–21], whereas lncXIST [22], lncNeat1 [23], and lncCRNDE [24] are reported to be associated with osteoclast differentiation. However, at present, there is insufficient information on specific lncRNAs involved in bone nonunion.

In this study, we obtained bone nonunion and union tissue samples from patient fracture sites. We performed transcriptome sequencing of these tissues to determine the differentially expressed lncRNAs (DELs) and differentially expressed mRNAs (DEGs) and identify potential lncRNA-mRNA interactions. Our findings may provide new insights to further elucidate the pathogenesis of, and develop biomarkers for, bone nonunion.

## 2. Methods

### 2.1. Sample Collection

The samples used in this study were obtained from three patients with normal fracture healing and three patients with bone nonunion (Table 1). The specimens were collected from the normal healing fracture site and scar tissue approximately 3 mm in size at the bone nonunion site. The diagnosis of bone nonunion was based on the definition given by the Food and Drug Administration. First, the fractures went unhealed for six months and there was no further healing trend within three months. Clinical X-ray examination was performed to confirm bone nonunion, and surgery further confirmed the formation of a small amount of scar tissue and callus at the fracture end or with only a small amount of scar tissue. None of the patients included in the study had infections, tumors, autoimmune diseases, bone nonunion caused by pathological fractures, history of hormone use, and history of smoking.

### 2.2. Total RNA Extraction

We extracted total RNA using TRIzol reagent following the manufacturer's protocol. The absorbance ratio at 260/280 nm (A260/A280) was measured using SmartSpec Plus to determine the concentration and purity of the isolated RNA. The integrity of the extracted RNA was confirmed using electrophoresis (1.5% agarose gel). The RNA was then transcribed into first-strand cDNA using the First-Strand cDNA Synthesis Kit (TaKaRa) for performing gene expression analysis.

### 2.3. lncRNA and mRNA Sequencing

We used 3 *μ*g RNA per sample for sample preparation. Following ribosomal RNA (rRNA) depletion, the RNA was fragmented and a cDNA library was constructed using the VAHTS Total RNA-seq (HMR) Library Prep Kit. Libraries were sequenced on an Illumina HiSeq 2500 platform according to the manufacturer's instructions, and 125 bp paired-end reads were produced (Table S1 & S2). DELs and DEGs between samples were identified using the Cuffdiff program in the Cufflinks package. As cutoff criteria, *p* values < 0.05 and |log2*FC*| > 1 were used.

### 2.4. Analysis of DELs and DEGs

Gene Ontology (GO) Enrichment and Kyoto Encyclopedia of Genes and Genomes (KEGG) pathway analysis for these DEGs and predicted target genes for DELs were conducted using the clusterProfiler R package. We obtained all the gene sets used in these functional annotations from the DAVID database. *p* values were adjusted by Benjamini & Hochberg methods, and FDR < 0.05 was defined as significantly enriched.

### 2.5. Prediction of Cis- and Trans-Regulated Target Genes of DELs

lncRNAs directly regulate adjacent target genes in the genome and this is termed cis-acting regulation. According to the taxonomic annotation information of lncRNA, neighboring known genes are predicted to be potential cis-regulated target genes. lncRNAs that are located far from their target genes play an indirect regulatory role through sequence complementarity, which is referred to as trans-acting regulation. We used RepeatMasker to search the *Alu* repeat structure of lncRNAs and 3′-UTRs. We used BLASTN sequence alignment to search for complementary sequence regions of lncRNAs and 3′-UTRs. The thermodynamic stability and binding ability of complexes formed by lncRNAs and 3′-UTRs were predicted by RNAplex and RIsearch, with an aim to predict trans-regulated target genes of lncRNAs.

## 3. Results

### 3.1. Boxplot and Principal Component Analysis (PCA) Diagram

**Figure 1:**
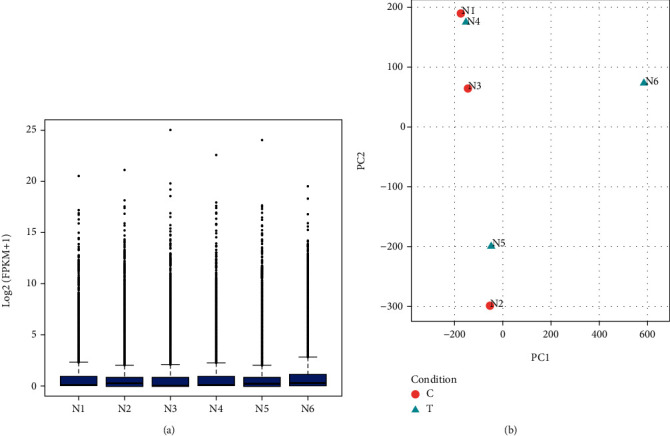
Transcript expression abundance and PCA plot of samples. (a) Boxplot of known transcript expression abundance; (b) PCA plot of the sample.

Boxplots describe data using five statistics, including the minimum, first quartile (25%), median (50%), third quartile (75%), and maximum. Through boxplots, we can gauge the symmetry of the data and the degree of dispersion of the distribution. As shown in Figure 1(a), we observed that the gene expression level of samples 1–5 was stable, while sample N6 was different, which may have been caused by a more serious fracture in patient 6.

It is possible to observe the similarity between samples through PCA plots. The closer the distance between samples on the PCA diagram, the closer the expression trend of sample genes is. As shown in Figure 1(b), the PCA diagram revealed that the expression features of samples 1–5 were similar, while sample N6 was different, which may be caused by a more serious fracture in patient 6.

### 3.2. Differential Expression of mRNAs and lncRNAs

**Figure 2:**
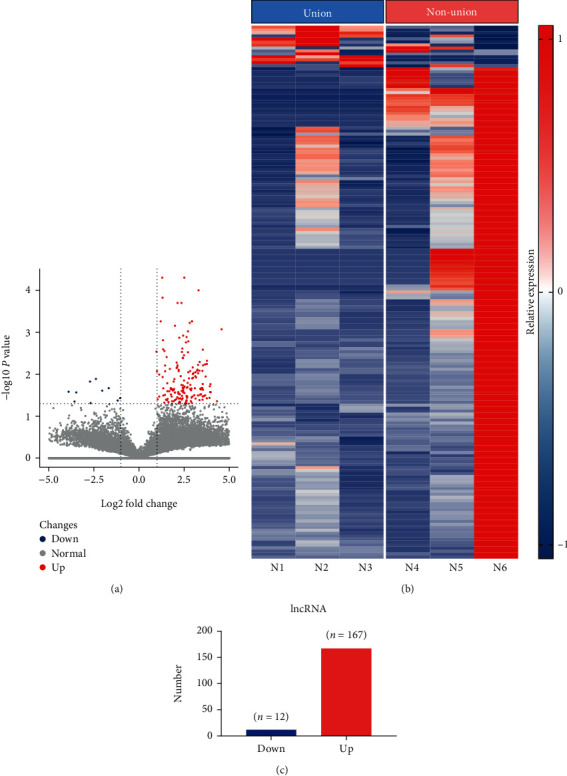
Differential expression of lncRNAs (DELs) between bone nonunion and union tissues. (a) Volcano plot showing DELs; (b) 179 DELs between bone nonunion and union group comprising 12 downregulated lncRNAs and 167 upregulated lncRNAs; (c) heatmap of DELs. T1, T2, and T3: bone union group; T4, T5, and T6: bone nonunion group.

**Figure 3:**
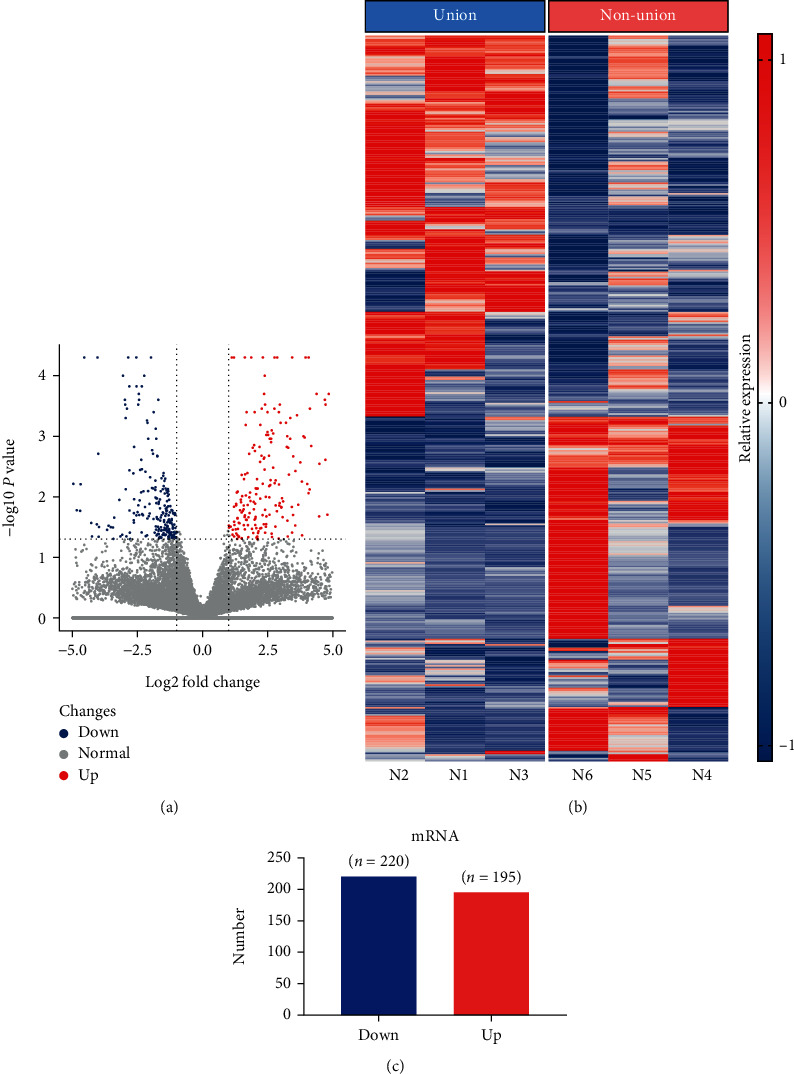
Differential expression of genes (DEGs) between bone nonunion and union tissues. (a) Volcano plot showing DEGs; (b) 415 DELs between bone nonunion and union group comprising 220 downregulated genes and 195 upregulated genes; (c) heatmap of DEGs. T1, T2, and T3: bone union group; T4, T5, and T6: bone nonunion group.

As shown in Figure 2, a volcano plot of DELs between normal fracture healing and bone nonunion tissue samples indicated 167 upregulated lncRNAs and 12 downregulated lncRNAs (Figures 2(a) and 2(b)). Additionally, 195 DEGs were upregulated and 220 DEGs were downregulated (Figures 3(a) and 3(b)). Figures 2(c) and 3(c) are cluster heatmaps of DELs and DEGs, respectively, indicating a large difference in the expression between normal fracture healing and bone nonunion tissue samples. The top 10 DELs and DEGs were indicated in Tables 2 and 3, respectively. Additionally, we annotated and classified the studied lncRNAs and they were mainly divided into intergenic lncRNAs, sense lncRNAs, intronic lncRNAs, antisense lncRNAs, sRNA host lncRNAs, enhancer lncRNAs, and bidirectional lncRNAs, accounting for 75.1%, 15.3%, 3.4%, 2.8%, 1.7%, and 0.6%, respectively (Figure S1).

### 3.3. Function and Pathway Predictive Analysis of DELs and DEGs

**Figure 4:**
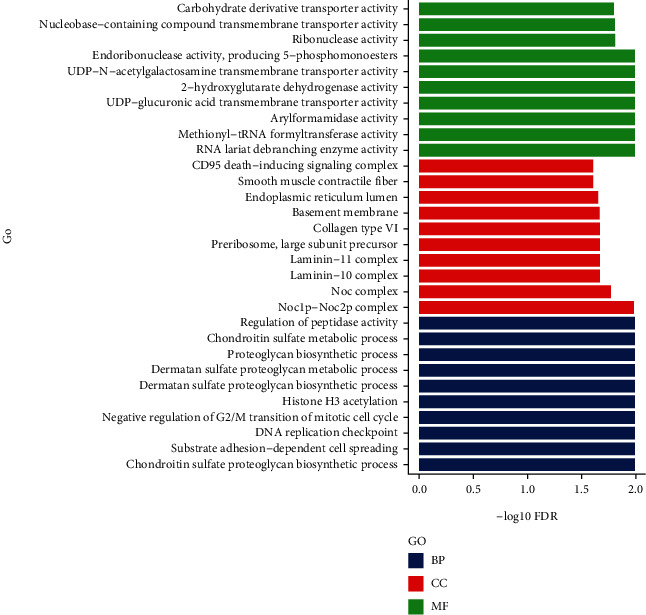
GO term enrichment analysis of DELs; the abscissa represented the −log10 FDR value and the ordinate represented the GO classification; green, red, and blue indicated molecular function (MF), cell component (CC), and biological process (BP), respectively.

The biological processes (BP), cellular components (CC), and molecular functions (MF) of DEGs and DELs were analyzed using the DAVID database. GO analysis of DELs in terms of MF showed carbohydrate derivative transporter activity and Se-containing compound transmembrane transporter activity as most enriched. GO analysis of DELs in terms of CC was enriched in the CD95 death-inducing signaling complex in cellular components and smooth muscle contractile fiber. GO analysis of DELs in terms of BP was predominantly enriched in the regulation of peptidase activity and dermatan sulfate proteoglycan metabolic process (Figure 4).

**Figure 5:**
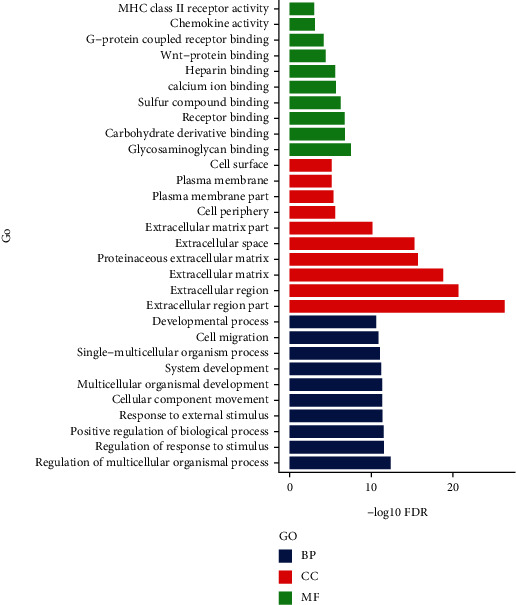
GO term enrichment analysis of (B) DEGs; the abscissa represented the −log10 FDR value and the ordinate represented the GO classification; green, red, and blue indicated molecular function (MF), cell component (CC), and biological process (BP), respectively.

GO analysis of DEGs for MF was primarily enriched in MHC class II receptor activity and chemokine activity, CC showed enrichment in cell surface and plasma membrane components, and BPs showed developmental processes and cell migration (Figure 5).

**Figure 6:**
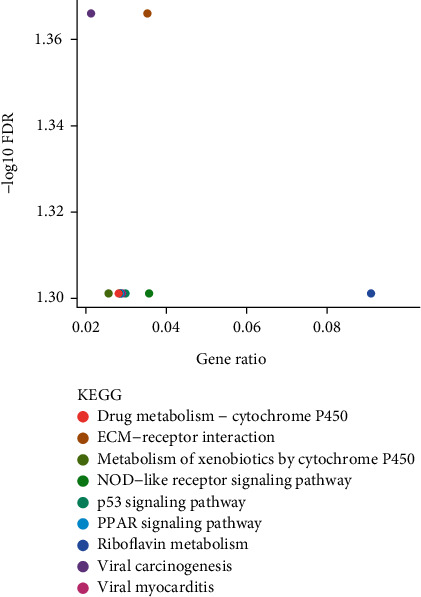
KEGG analysis of the potential pathway enriched by DELs; the abscissa represented the gene ratio and the ordinate represented the −log10 FDR value.

**Figure 7:**
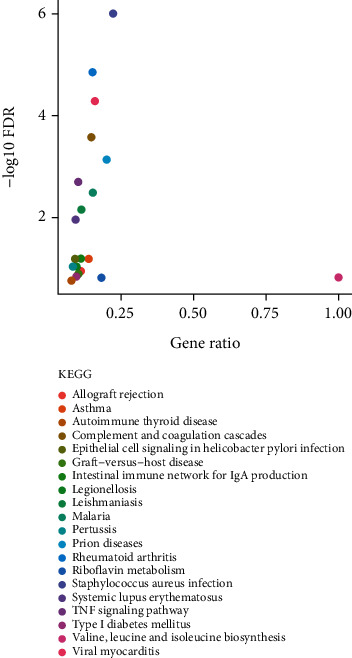
KEGG analysis of the potential pathway enriched by DEGs; the abscissa represented the gene ratio and the ordinate represented the −log10 FDR value.

**Figure 8:**
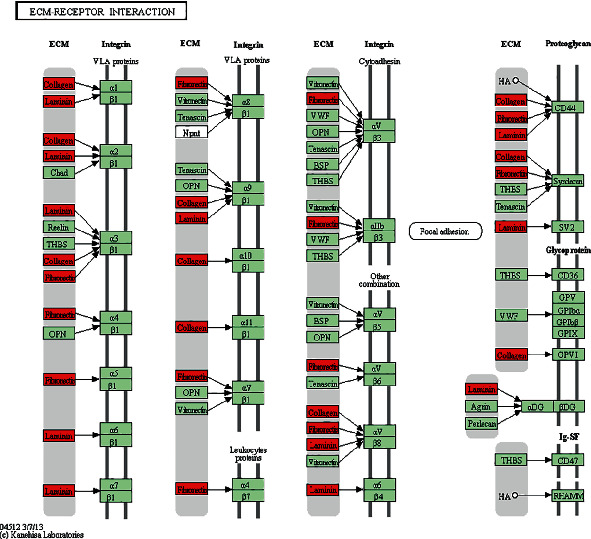
KEGG pathway of ECM-receptor interaction. Red indicated significantly different expression genes in the bone nonunion group compared with bone union group. Organism-specific genes or pathways were colored green.

**Figure 9:**
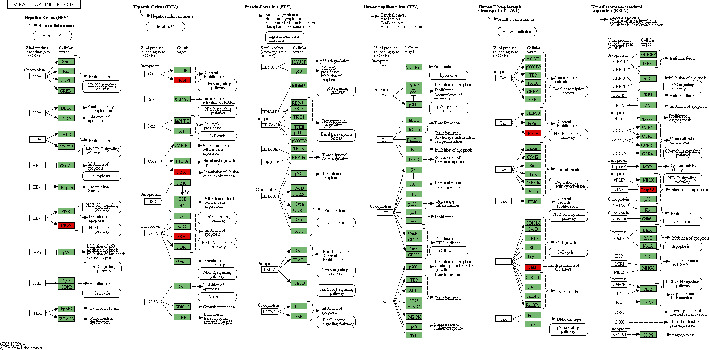
KEGG pathway of viral carcinogenesis. Red indicated significantly different expression genes in the bone nonunion group compared with the bone union group. Organism-specific genes or pathways were colored green.

**Figure 10:**
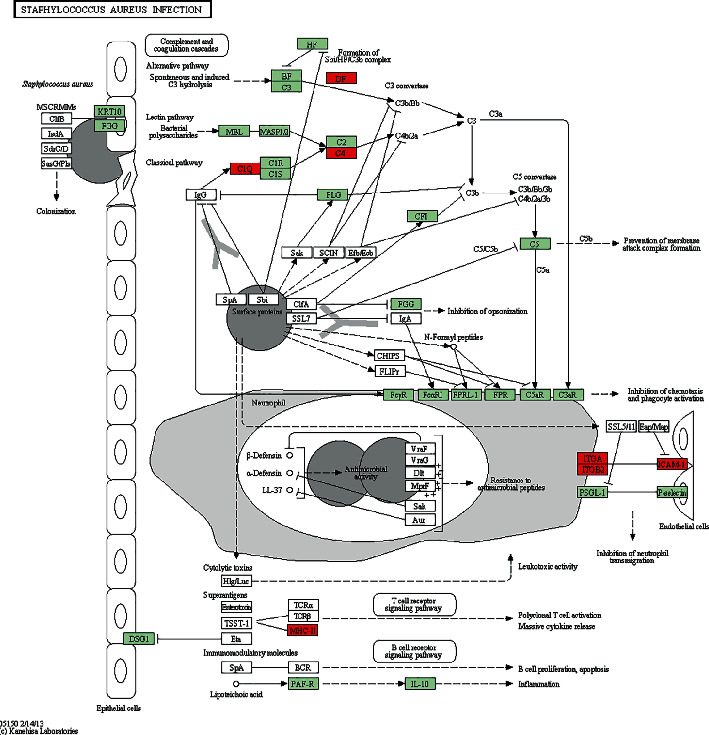
KEGG pathway of staphylococcus aureus infection. Red indicated significantly different expression genes in the bone nonunion group compared with the bone union group. Organism-specific genes or pathways were colored green.

**Figure 11:**
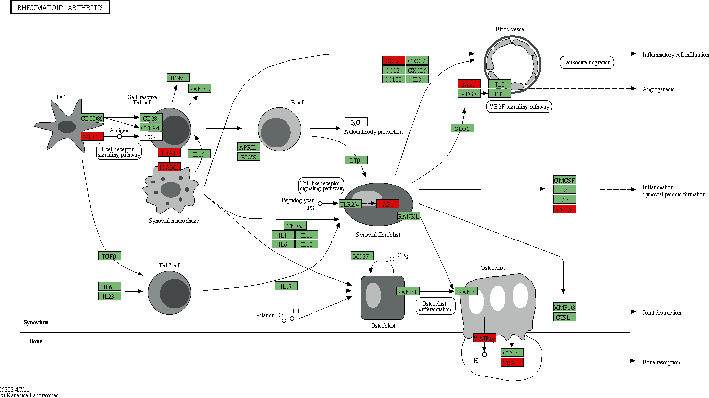
KEGG pathway of rheumatoid arthritis. Red indicated significantly different expression genes in the bone nonunion group compared with the bone union group. Organism-specific genes or pathways were colored green.

The KEGG analyses for DELs (Figure 6) and DEGs (Figure 7) were also performed. The significant KEGG functional enrichment of DELs was ECM-receptor interaction (Figure 8), viral carcinogenesis (Figure 9), drug metabolism, cytochrome P450, p53 signaling pathway, nucleotide-binding oligomerization domain- (NOD-) like receptor signaling pathway, viral myocarditis, peroxisome proliferator-activated receptor (PPAR) signaling pathway, metabolism of xenobiotics by cytochrome P450, and riboflavin metabolism. The significant KEGG functional enrichment of DEGs was valine, leucine and isoleucine biosynthesis, *Staphylococcus aureus* infection (Figure 10), prion diseases, riboflavin metabolism, viral myocarditis, malaria, rheumatoid arthritis (Figure 11), complement and coagulation cascades, asthma, legionellosis, intestinal immune network for IgA production, allograft rejection, graft-versus-host disease, tumor necrosis factor (TNF) signaling pathway, legionellosis, epithelial cell signaling in *Helicobacter pylori* infection, type I diabetes mellitus, systemic lupus erythematosus, pertussis, and autoimmune thyroid disease.

### 3.4. lncRNA Cis- and Trans-Regulated Genes

**Figure 12:**
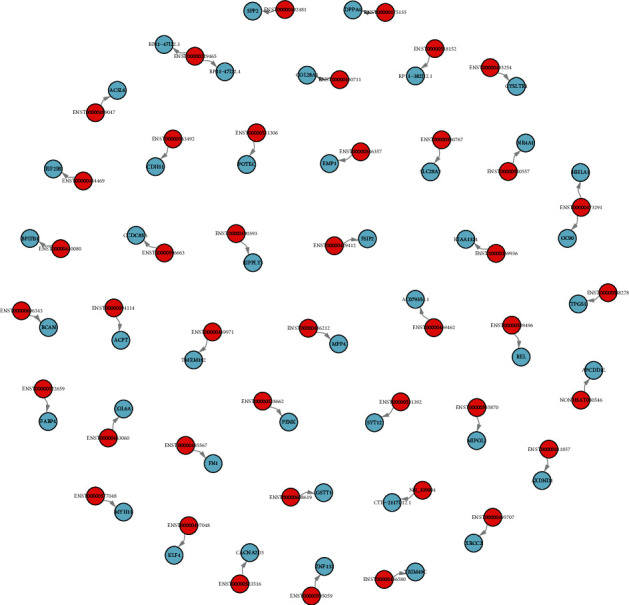
The cis-regulated target gene network diagram of differentially expressed lncRNAs. Red represented lncRNA and blue represented the target gene.

**Figure 13:**
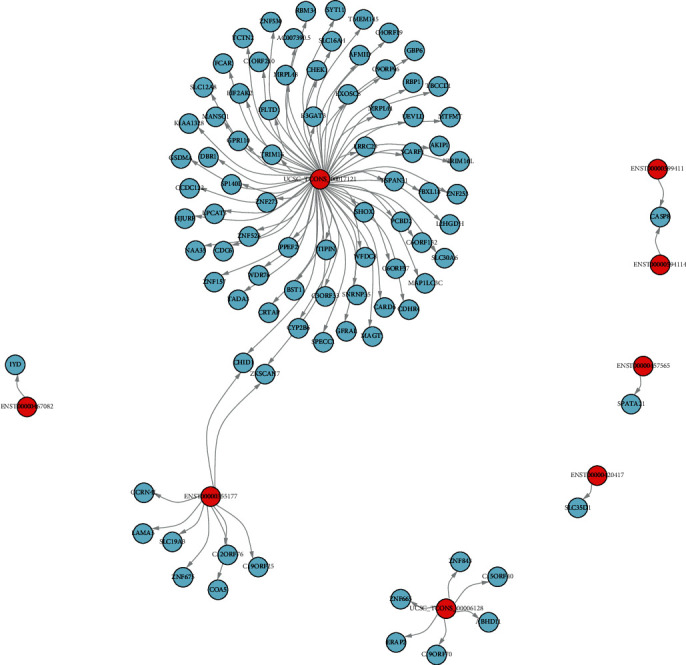
The trans-regulated target gene network diagram of differentially expressed lncRNAs. Red represented lncRNA and blue the represented target gene.

Differential expression of lncRNA cis- and trans-regulated genes was predicted. As shown in Figure 6, lncRNA ENST00000453060 cis-regulates COL6A1, lncRNA ENST00000577048 cis-regulates MYH11, lncRNA ENST00000606343 cis-regulates BCAN, etc. (Figure 12). Additionally, several lncRNA trans-regulated genes were reported, including lncRNA UCSC_TCONS_00017121 trans-regulating RBP1, MLPL44, MTFMT, and TIPIN (Figure 13).

## 4. Discussion

With the development of sequencing technology, transcriptome sequencing has been used to understand a variety of diseases [25, 26]. Wei et al. used an miRNA expression profile of bone nonunion and union tissues to find nine upregulated and nine downregulated miRNAs [27]. Long et al. reported 557 differentially expressed miRNAs in bone nonunion tissues and further explored that miR-381 can modulate human bone mesenchymal stromal cell osteogenesis [28]. The results obtained in different transcriptome sequencing studies may vary greatly, which may be related to different sequencing technologies, samples, and sequencing methods. Previous studies indicated that lncRNAs achieve their functions in tumors through a wide range of mechanisms [29–31]. However, lncRNAs have been rarely studied in orthopedics, especially with respect to bone nonunion [32], thus limiting the detection and treatment of bone nonunion to a certain extent.

In this study, transcriptome sequencing was performed on bone tissue samples collected from long bones (tibia, femur, and humerus) of patients with bone nonunion and normal bone union. We detected and analyzed 179 DELs and 415 DEGs. GO analysis showed that DELs were primarily enriched in carbohydrate derivative transporter activity in MF, CD95 death-inducing signaling complex in CC, and regulation of peptidase activity in BP. The DEGs were mainly involved in MHC class II receptor activity for MF, cell surface, and developmental processes for CC and BP. The KEGG pathway enrichment of the DELs showed the ECM-receptor interaction pathway and viral carcinogenesis pathway. The KEGG pathway enrichment in DEGs showed the *S. aureus* infection pathway and rheumatoid arthritis pathway. The ECM-receptor interaction pathway primarily functions through three ECM proteins, including collagen, fibronectin, and laminin. Laminin is involved in osteogenesis and promotion of bone defect repair [33, 34]. Studies have suggested that collagen type XV may be involved in ECM organization early in the osteogenesis process, a prerequisite for promoting subsequent mineral matrix deposition [35]. Immunohistochemical and transcriptomic studies have shown the expression and dynamic regulation of fibronectin in several stages of fracture healing [35, 36]. Single-cell RNA sequencing of the injury site revealed an early increase in mesenchymal progenitor cell (MPC) genes associated with cell adhesion pathways and ECM receptor interactions. The ECM creates a microenvironment with a MPC differentiation bias closer to a specific stiffness role in tissues [37–40]. For example, a rigid environment that mimics the natural bone favors differentiation into osteoblasts [41], whereas a softer matrix promotes the development of adipocyte fate [42, 43].

lncRNAs can cis-regulate the transcription of adjacent protein-coding genes, thereby regulating the expression of such genes and participating in developmental and other biological processes associated with them. Cis-regulation refers to the transcriptional activation and expression regulation of noncoding RNAs to adjacent mRNAs. In this study, we found that lncRNA ENST00000453060 may cis-regulate COL6A1. Previous studies have indicated that genetic deletion of COL6A1 impairs osteoblast connections and communication [44]. COL6A1 plays a substantial role in osteoblasts, and lncRNA ENST00000453060 may regulate osteoblasts via cis-regulation of COL6A1. Additionally, our previous study confirmed that lncRNA ENST00000563492 could promote the osteogenesis-angiogenesis coupling process in bone marrow stromal cells [45].

lncRNA trans-regulation is the regulation of distal mRNA transcription. lncRNAs can regulate the expression of distant genes by binding to enhancers and promoters. lncRNAs regulate the activity of bound proteins or RNAs in the cytoplasm or nucleus in a dose-dependent manner. The lncRNA UCSC_TCONS_00017121 trans-regulates RBP1. Previous studies have shown that RBP1 promotes differentiation of osteoblasts [46]. The lncRNA UCSC_TCONS_00017121 may also regulate osteoblasts via cis-regulating COL6A1.

There were certain limitations in the present study. First, no validation assays, including qPCR and histological analysis, were performed to confirm the differential expression, thereby demanding the need for further experimental studies. Second, patient matching, including differences in ages of enrolled patients and differences involving sites of sample collection from bone nonunion tissues, was not well handled. Third, only 6 patients were enrolled in this study, the results from which need to be confirmed in a further study with greater numbers of samples. Fourth, the sample size of this study was relatively small and the results need to be interpreted with caution.

## 5. Conclusions

A total of 179 DELs and 415 DEGs were identified between bone nonunion and bone union tissue samples. All of these lncRNAs and mRNAs may be related to the occurrence and development of bone nonunion. GO, KEGG, and regulatory analysis for these lncRNAs and mRNAs were performed to detect their potential functions. This study identified potential biomarkers for bone nonunion, but a validation cohort is still essential to confirm the applicability of these biomarkers.

## Figures and Tables

**Table 1:** Basic characteristics of enrolled patients.

Sample	Sex	Age (years)	Location	Healing status
No. 1	Male	41	Left humerus	Union
No. 2	Male	45	Right tibia	Union
No. 3	Male	24	Right femur	Union
No. 4	Male	47	Right humerus	Nonunion
No. 5	Male	46	Left tibia	Nonunion
No. 6	Male	25	Left femur	Nonunion

**Table 2:** Top 10 differentially expressed lncRNAs.

Gene ID	Gene	Locus	log2 (fold change)	*p* value
*Upregulated lncRNAs*				
ENST00000485567	FN1	2:216225162-216300895	30.494	0.0017
ENST00000605228	RP1	1:182403027-182403596	5.023756571	0.00845
ENST00000603389	WI2	2:16330518-16330662	4.381871684	0.044
ENST00000452690	RP11	X:40122130-40146973	3.95783	0.01945
ENST00000420417	RP11	8:99973655-99980512	3.94771	0.0366
ENST00000366224	RP11	X:47157250-47158120	3.9219	0.02685
ENST00000331301	AP002387.1	11:71093646-71134469	3.90025	0.02675
ENST00000550756	OLA1P3	12:56263831-56266386	3.88772	0.0173
ENST00000475135	DPPA4	3:109044987-109056419	3.79153	0.0377
ENST00000603371	RP11	X:35882974-35887748	3.78534	0.0057
*Down-regulated lncRNAs*				
ENST00000463060	COL6A1	21:47401650-47424964	−33.8802	0.00215
ENST00000546357	EMP1	12:13349649-13369708	−11.3747	0.0211
ENST00000577048	AF001548.6	16:15005407-16444465	−3.90021	0.0262
ENST00000563492	CDH11	16:64977655-65160015	−3.54485	0.04475
ENST00000522659	FABP4	8:82351670-82445510	−3.4878	0.0273
ENST00000573866	SNORD3D	17:19015312-19015949	−2.80835	0.01495
ENST00000550557	NR4A1	12:52416615-52453291	−2.6977	0.04875
ENST00000497048	KLF4	9:110247132-110252763	−2.3589	0.01295
ENST00000566457	CTD	8:22532053-22541522	−1.9616	0.0247
ENST00000569449	RP11	4:156655599-156658214	−1.05101	0.0372

**Table 3:** Top 10 differentially expressed genes.

Gene ID	Gene	Locus	log2 (fold change)	*p* value
*Upregulated genes*				
ENST00000457143	ATP5J	21:26931715-27589700	45.5374	0.04015
ENST00000539409	FAM60A	12:31433517-31479992	11.9547	0.03395
ENST00000401325	AC009695.1	8:21351538-21351627	11.40504571	0.044
ENST00000582836	AC003035.1	X:14093909-14094010	10.67193906	0.044
ENST00000365209	Y_RNA	1:247458136-247458243	9.618411199	0.044
ENST00000363299	RNU5D-1	1:45196726-45196842	8.430795412	0.03395
ENST00000435777	COL22A1	8:139600477-139926249	5.3695	0.0358
ENST00000226284	IBSP	4:88720732-88733074	4.97665	5.00*E* − 05
ENST00000324559	ANO5	11:22214721-22304903	4.81686	0.0002
ENST00000361131	PPP1R14C	6:150464211-150571493	4.57736	0.00285
*Down-regulated genes*				
ENST00000505243	RPS3A	4:152020724-152246795	−142.886	0.0414
ENST00000502527	VCAN	5:82767283-82878122	−17.498	0.015
ENST00000455022	UTRN	6:144606290-145174170	−13.8992	0.0163
ENST00000390603	IGHV3-15	14:105992939-107283280	−5.23362	0.00945
ENST00000512158	CXCL14	5:134895266-134970564	−5.00246	0.02945
ENST00000326245	ITLN1	1:160846328-160854960	−4.95648	0.0061
ENST00000390600	IGHV3-9	14:105992939-107283280	−4.88897	0.01665
ENST00000492446	IGKV1D-16	2:90139077-90139580	−4.76505	0.01705
ENST00000390598	IGHV3-7	14:105992939-107283280	−4.73485	0.0062
ENST00000343267	APOD	3:195295572-195311076	−4.44718	5.00*E* − 05

## Data Availability

The datasets used and/or analyzed during the current study are available from the corresponding author upon reasonable request.

## Supplementary Materials

Supplementary Materials Figure S1: annotation and classification of the lncRNAs obtained. Table S1: statistical results of original and preprocessed sequences. Table S2: statistical results of reference genome alignment analysis of reads.
