# Supplementary material for: Transcriptome Sequencing Analysis of lncRNA and mRNA Expression Profiles in Bone Nonunion
Source: Oxid Med Cell Longev. 2022 Oct 12;2022:9110449. doi: 10.1155/2022/9110449 (PMC9581694; doi:10.1155/2022/9110449)
Supplement: Supplementary Materials — Figure S1: annotation and classification of the lncRNAs obtained. Table S1: statistical results of original and preprocessed sequences. Table S2: statistical results of reference genome alignment analysis of reads. [file 9110449.f1.doc]

**Supplementary materials**


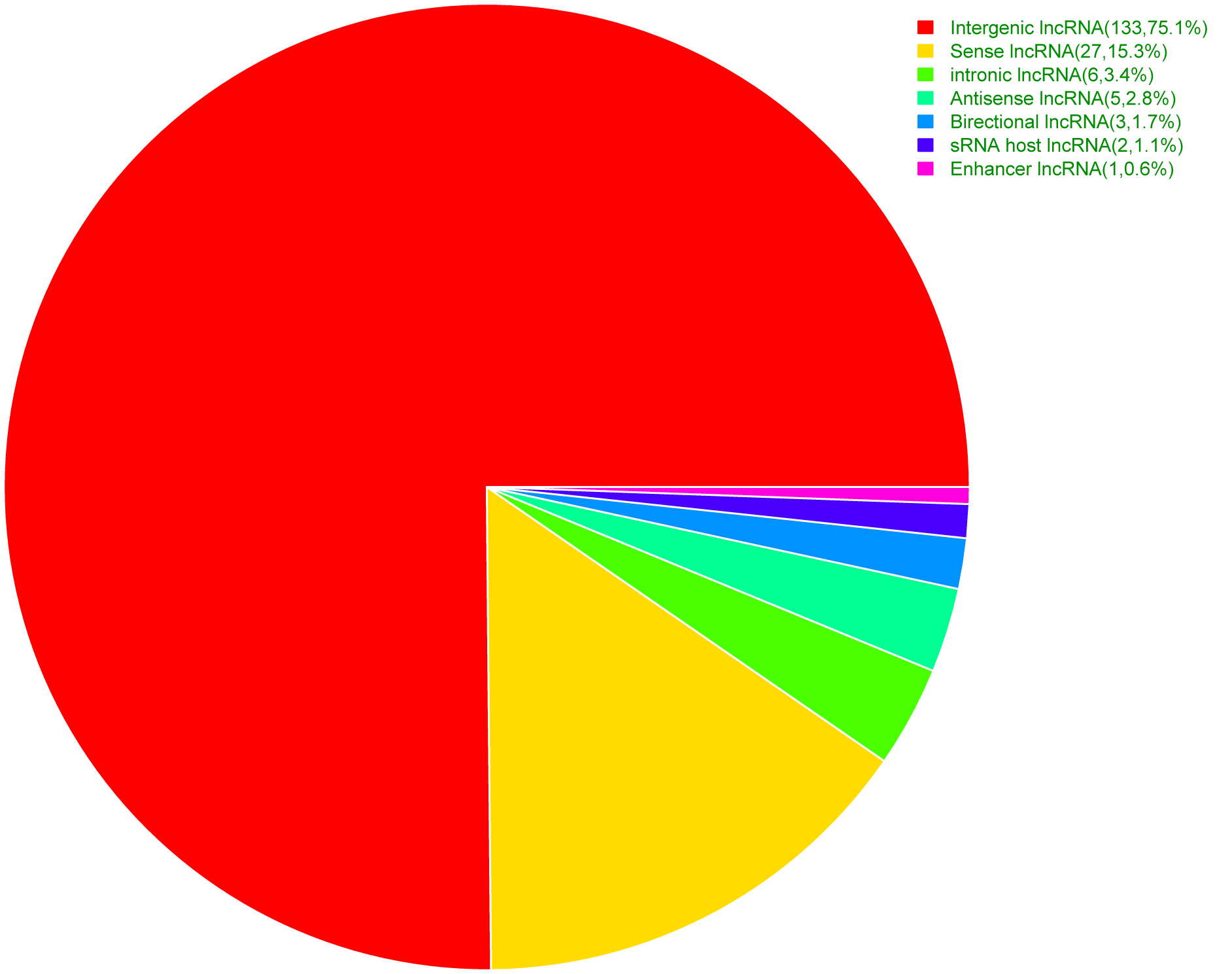
Figure S1. Annotation and classification of the lncRNAs obtained.

Table S1. Statistical results of original and preprocessed sequences.

| Sample | Healing status | Raw reads | Raw read pairs | Raw bases | Trim reads | Trim bases | Trim read pairs | Average length | Trim reads % | Trim bases % |
| --- | --- | --- | --- | --- | --- | --- | --- | --- | --- | --- |
| NO. 1 | bone union | 63592624 | 6422855024 | 31796312 | 60009178 | 5858689159 | 30004589 | 97.6 | 0.944 | 0.912 |
| NO. 2 | bone union | 84197564 | 42098782 | 8503953964 | 79772396 | 7764964879 | 39886198 | 97.3389953 | 0.94744304 | 0.913100531 |
| NO. 3 | bone union | 86143456 | 43071728 | 8700489056 | 79200308 | 7573743727 | 39600154 | 95.62770548 | 0.919400169 | 0.870496322 |
| NO. 4 | bone nonunion | 61015766 | 6162592366 | 30507883 | 57814086 | 5658758559 | 28907043 | 97.9 | 0.948 | 0.918 |
| NO. 5 | bone nonunion | 87411498 | 43705749 | 8828561298 | 81056088 | 7785670188 | 40528044 | 96.05287376 | 0.927293203 | 0.88187304 |
| NO. 6 | bone nonunion | 75476226 | 37738113 | 7623098826 | 71262700 | 6951347258 | 35631350 | 97.5453815 | 0.94417413 | 0.911879462 |

Table S2. Statistical results of reference genome alignment analysis of reads.

| Sample | Total reads | Total mapped | Mapped ratio(%) | Multiple mapped | Unique mapped | Read-1 | Read-2 | Reads map to '+' | Reads map to '-' | Non-Splice reads | Splice reads | Reads Proper pair |
| --- | --- | --- | --- | --- | --- | --- | --- | --- | --- | --- | --- | --- |
| N1 | 60009178 | 53567358 | 89.30% | 2346598 | 51220760 | 25588165 | 25632595 | 25575316 | 25645444 | 43880559 | 7340201 | 44134972 |
| N2 | 79772396 | 67213951 | 84.30% | 3337569 | 63876382 | 32112286 | 31764096 | 31905830 | 31970552 | 58058303 | 5818079 | 52868082 |
| N3 | 79200308 | 40654684 | 51.30% | 2193985 | 38460699 | 19122061 | 19338638 | 19030101 | 19430598 | 35262798 | 3197901 | 28916912 |
| N4 | 57814086 | 50280890 | 87% | 2340428 | 47940462 | 23936665 | 24003797 | 23842078 | 24098384 | 40869064 | 7071398 | 40142480 |
| N5 | 81056088 | 55014387 | 67.90% | 2770722 | 52243665 | 26141553 | 26102112 | 26389354 | 25854311 | 47551850 | 4691815 | 40519114 |
| N6 | 71262700 | 47730421 | 67% | 3113254 | 44617167 | 24048223 | 20568944 | 22300278 | 22316889 | 43450280 | 1166887 | 23402534 |
